# Conceptual Links between Landscape Diversity and Diet Diversity: A Roadmap for Transdisciplinary Research

**DOI:** 10.1093/biosci/biaa048

**Published:** 2020-06-24

**Authors:** Sarah E Gergel, Bronwen Powell, FrÉdÉric Baudron, Sylvia L R Wood, Jeanine M Rhemtulla, Gina Kennedy, Laura V Rasmussen, Amy Ickowitz, Matthew E Fagan, Erica A H Smithwick, Jessica Ranieri, Stephen A Wood, Jeroen C J Groot, Terry C H Sunderland

**Affiliations:** Department of Forest and Conservation Sciences, University of British Columbia, Vancouver, Canada; Department of Geography and BP is also affiliated with the Departments of African Studies and Anthropology at Pennsylvania State University, University Park, Pennsylvania; International Maize and Wheat Improvement Center CIMMYT-Southern Africa Regional Office, Harare, Zimbabwe; Future Earth Global Hub, Montreal, Quebec, Canada; Department of Forest and Conservation Sciences, University of British Columbia, Vancouver, Canada; Bioversity International, Rome, Italy; Department of Forest and Conservation Sciences, University of British Columbia, Vancouver, Canada; Center for International Forestry Research, Bogor, Indonesia; Department of Geography and Environmental Systems, University of Maryland—Baltimore County, Baltimore, Maryland; Department of Geography and BP is also affiliated with the Departments of African Studies and Anthropology at Pennsylvania State University, University Park, Pennsylvania; Bioversity International, Rome, Italy; Nature Conservancy, Arlington, Virginia, and with the School of Forestry and Environmental Studies, Yale University, New Haven, Connecticut; Department of Farming Systems Ecology, Wageningen University and Research, Wageningen, The Netherlands; Department of Forest and Conservation Sciences, University of British Columbia, Vancouver, Canada; Center for International Forestry Research, Bogor, Indonesia

**Keywords:** sustainable development, landscape approach, food security and nutrition, remote sensing, tropical forest conservation and restoration

## Abstract

Malnutrition linked to poor quality diets affects at least 2 billion people. Forests, as well as agricultural systems linked to trees, are key sources of dietary diversity in rural settings. In the present article, we develop conceptual links between diet diversity and forested landscape mosaics within the rural tropics. First, we summarize the state of knowledge regarding diets obtained from forests, trees, and agroforests. We then hypothesize how disturbed secondary forests, edge habitats, forest access, and landscape diversity can function in bolstering dietary diversity. Taken together, these ideas help us build a framework illuminating four pathways (direct, agroecological, energy, and market pathways) connecting forested landscapes to diet diversity. Finally, we offer recommendations to fill remaining knowledge gaps related to diet and forest cover monitoring. We argue that better evaluation of the role of land cover complexity will help avoid overly simplistic views of food security and, instead, uncover nutritional synergies with forest conservation and restoration.

Over two billion people suffer from deficiencies in essential vitamins and minerals, a problem known as hidden hunger (Development Initiatives 2018). Improving diet quality is part of overcoming micronutrient deficiencies while also contributing to better health outcomes. Poor quality diets (such as low consumption of whole grains, fruits and vegetables, or high consumption of red meat, processed foods, salt, fat, and/or added sugars) are associated with higher risk for many chronic diseases and are now among the leading modifiable risk factors for mortality globally (Development Initiatives 2018, Afshin et al. 2019).

Dietary diversity is associated with higher dietary quality (Ruel 2003, Kennedy et al. 2011). When considering not just calories alone but diet quality and diversity, forests are an important contributor to human diets (Ickowitz et al. 2014, Rowland et al. 2017), particularly for those living in proximity to forests. For example, forests and trees are sources of several food groups containing micronutrients of global nutrition concern including iron, zinc, vitamin A, and folate (Powell et al. 2013a). These food groups include dark green leafy vegetables, fruits, and meat. A growing body of evidence links tree cover (i.e., the percentage of the land area under tree canopy) to dietary quality and diversity, along with other indices of nutrition (Johnson et al. 2013, Ickowitz et al. 2014, Galway et al. 2018, Rasolofoson et al. 2018, Hall et al. 2019, Lo et al. 2019). For example, across 21 countries, Ickowitz and colleagues (2014) demonstrated that the dietary diversity of children was positively correlated with the percentage of tree cover surrounding their communities. For 27 developing countries, Rasolofoson and colleagues (2018) estimated that living in highly forested areas increased the dietary diversity of children by 25% compared with those in less forested areas. Across 15 African countries, Galway and colleagues (2018) showed that child dietary diversity was negatively correlated with forest loss. However, the mechanistic pathways explaining these relationships remain poorly resolved.

In current debates about how to feed the world's growing population, a focus on yields and calories has placed a pervasive emphasis on agriculture, livestock, and fisheries (Ickowitz et al. 2019). We argue that this has contributed to blind spots in the role of landscape diversity, especially where agriculture is situated within a mosaic of trees and forest. Although ten crops account for two-thirds of global cropland (Dawson et al. 2019), their perceived advantage relies heavily on measures of yield and less on nutrition (Remans et al. 2014, DeFries et al. 2015). Such a singular focus on agricultural yields and calories arguably oversimplifies food–forest–conservation debates. As a result, forests and trees are rarely integrated into food security, nutrition, and agricultural development strategies (Ruel and Alderman 2013, HLPE 2017, Downs et al. 2020). We posit that diet and nutrition, particularly dietary diversity, can benefit from a broader landscape perspective that not only addresses agriculture but also integrates forest conservation and restoration. Approaches that place dietary quality and nutrition more centrally and that seek to understand diet–landscape relationships are integral to meeting twenty-first century nutrition and food security goals, especially in many low-income rural regions of the world (Remans et al. 2011, DeFries et al. 2015, Powell et al. 2015, Ickowitz et al. 2019, Sunderland et al. 2019).

In the present article, we aim to foster the integration of forests into strategic thinking about agriculture, nutrition, and food security in rural tropical regions. To do so, we first explore the empirical basis of how forests, tree cover, and landscape diversity help support dietary diversity. We then identify remaining knowledge gaps with respect to the role of landscape diversity in enhancing dietary diversity. Finally, to strengthen research, we present a conceptual framework for guiding synthesis on the role of forests and diverse landscapes in enhancing dietary diversity. In addition to filling this conceptual gap, we also propose ways to fill remaining knowledge gaps through enhanced monitoring of forests and diets. We conclude by connecting our perspectives to synergistic outcomes for conservation and forest restoration. Taken together, we frame a comprehensive research agenda to help answer the question How might forests and landscape diversity support dietary diversity?

## Empirical basis for the role of forests and trees in dietary diversity

### Forest foods

**Figure 1.**
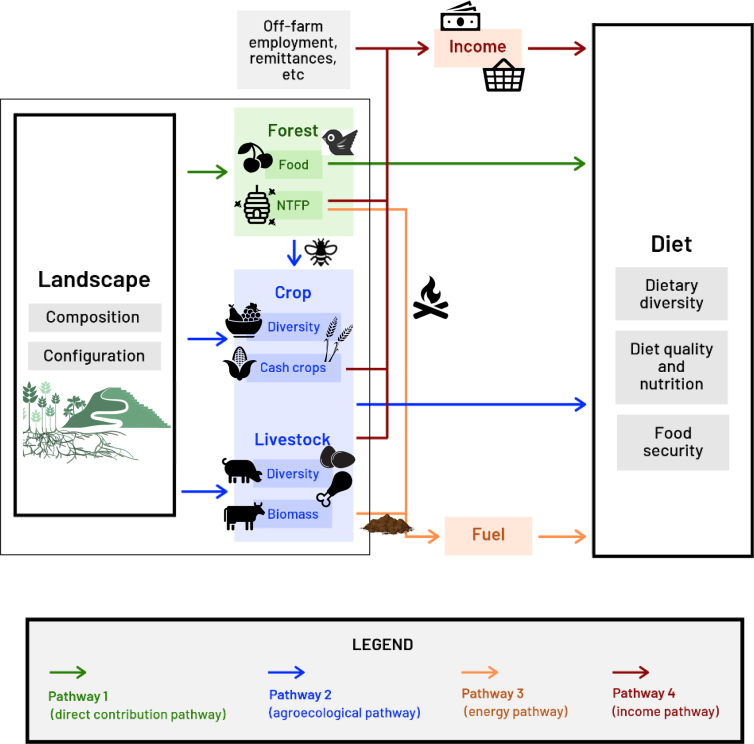
Landscape diversity can contribute to dietary diversity through four complex interacting pathways. Although forests make direct contributions to diets, landscape mosaics composed of forests and agriculture also interact to contribute to dietary diversity through several indirect pathways. The direct forest pathway can be critical during seasonal lean periods for agriculture and can provide income that enables purchase from markets. Market access can result in both beneficial and detrimental impacts on the quality of human diets. In an ideal situation for nutrition and dietary diversity, markets enable purchase of diverse nutritious foods. In a less than ideal situation, landscapes producing only a few commercial crops can give rise to local markets with fewer fresh foods and more highly processed, less healthy foods.

Significant evidence is emerging that forests, agroforestry systems, home gardens, and trees on farms provide nutritional benefits to millions of people, complementing other agricultural production systems (Kumar 2006, Powell et al. 2013a). Forests—defined as areas with tree canopy cover exceeding 10% and larger than 0.5 hectares (FAO 2010)—contribute to nutrition through a variety of direct and indirect mechanisms that have only recently become more widely acknowledged (HLPE 2017). Forests contribute directly to diverse, nutritious diets (pathway 1 in figure 1) by serving as a source of wild foods, the most commonly consumed being vegetables, mushrooms, fruit, insects, and wild meat, including birds and fish (Boedecker et al. 2014, Powell et al. 2015, Tata et al. 2019). Forests also contain habitat for a variety of wildlife species (bushmeat and fish) that provide critical micronutrients (e.g., iron; Fa et al. 2003, Blaney et al. 2009, Golden et al. 2011, Nasi et al. 2011, Lo et al. 2019). Furthermore, women rely on forest products differently from how men do (Sunderland et al. 2014), with far-reaching effects on household diets, given women's decision-making and control over food provisioning (Herforth and Ahmed 2015, Malapit and Quisumbing 2015).

### Tree-based agricultural systems

Agroforestry is the deliberate retention or integration of trees on farms, either alongside crops or in rotation (Leakey 1996). Trees in agroforestry systems produce food directly (via fruits and nuts) but also support the productivity of crops and livestock via ecosystem service benefits (Reed et al. 2017) that include moderating harsh microclimates (Sida et al. 2018a), promoting the return of organic matter to soils (via litterfall and root turnover), and improving soil fertility (Kumar 2006). Deep tree roots can also mobilize nutrients and access water deep below ground and can reduce erosion (Garrity 2004, Kumar 2006, Jamnadass et al. 2013, Zomer et al. 2014). Forests and trees in agricultural systems also provide habitats for natural enemies of crop and livestock pests (Bianchi et al. 2006). As a result of these types of regulating and supporting ecosystem services (pathway 2 in figure 1), some crops grown in agroforestry combinations achieve higher yields (Kumar 2006, Sida et al. 2018a). And further, soils with increased nutrients can translate into micronutrients in food (Frossard et al. 2000, Lal 2009, Arhin and Kazapoe 2017, Wood et al. 2018). The inclusion of fruit trees in agroforestry systems is also important to improving fruit consumption (McMullin et al. 2019).

Home gardens are small plots of cultivated land typically located close to the homestead (Powell et al. 2015). They often include overstory trees, crops, and a mix of wild and semidomesticated species (Freedman 2015). The biotic diversity of home gardens, along with their close proximity to homesteads, makes them an important source of nutritionally important food (Kumar and Nair 2004, Powell et al. 2015). Four separate reviews of the affects of agricultural interventions on nutrition outcomes (Tontisirin et al. 2002, Berti et al. 2004, Girard et al. 2012, Masset et al. 2012) each noted that home garden interventions are one of the most successful types of agricultural interventions for improving diet and nutrition (Powell et al. 2015). The type and diversity of home gardens have been found to be more important for diet quality than the size of the garden (Bloem et al. 1996). Home gardens are particularity important in marginal arid lands, which are home to 33% of the global population (Hori et al. 2012). In such landscapes, the nutrient flux from surrounding forests and trees not only maintains agriculture (such as row crops and livestock) but can also provide nutrient inputs that support home garden soils (Baudron et al. 2017).

### Landscape diversity

Despite their nutritional advantages, many diverse agriculture systems that include trees are increasingly being replaced by commercial monocultures (Kumar and Nair 2004, Padoch and Sunderland 2014), with complex effects on nutrition. In some regions, access to cash income enables households to purchase foods that diversify their diets (Sibhatu et al. 2015, Remans et al. 2014). Also, a number of studies have shown market access to be associated with greater dietary diversity (Sibhatu et al. 2015, Jones 2017). However, higher household cash flows may also be associated with more numerous, frequent, and larger-quantity purchases of highly processed and micronutrient-poor foods (Popkin 2004, Reyes-Garcia et al. 2019). For example, in Ghana, the introduction of commercial cacao production was associated with lower nutritional diversity (Anderman et al. 2014, Remans et al. 2014). As such, a view toward the interaction of forests with various forms of agriculture nearby is needed.

Landscape diversity—the number and types of different land cover and their spatial distribution (Gergel and Turner 2017)—is an emerging and essential component of ­nutrition-sensitive landscapes (Powell et al. 2013a). Nutrition interventions alone, such as supplementation and fortification of single nutrients in single crops, cannot meet global targets for reducing all forms of malnutrition. This has led to increasing calls for cross-sectoral attention to nutrition and diet quality, especially in agriculture (Ruel and Alderman 2013). As a result, nutrition-sensitive landscapes are gaining attention, with the goal of building ecological and nutritional diversity into landscape policy and planning (Powell et al. 2013a). The approach aspires to provide multiple sources of nutrients to people along with other ecosystem services (Reed et al. 2017). To achieve this goal, nutrition would be necessarily integrated into policies and programs that are also cognizant of environmental targets. Wild forest foods, for example, would be assessed in hunger and poverty alleviation programs, as well as in protected area management. However, this new appreciation of food–­forest–landscape dynamics lacks a full understanding of how various configurations of forests, trees, and fields interact to buoy dietary diversity (Rasmussen et al. 2019).

We argue that operationalizing the concept of landscape diversity with regards to diet and nutrition is underdeveloped from both conceptual and technical perspectives. In the present article, taking a broader landscape perspective, we place particular emphasis on the availability of and access to forests and trees, as well as their type, stand age, and travel distance, all of which potentially influence the ways forests and trees affect diverse diets. Concepts and approaches from landscape ecology and spatial analysis are well suited for providing insights into how landscape structure and configuration can support dietary diversity.

## Food for thought: Hypotheses to deepen our understanding of the nutritional function of landscapes

Building on the evidence base above, we further integrate landscape ecological principles into four landscape-level hypotheses (H1–H4) that provide a rich arena for additional research, refinement, and evaluation.

Disturbed and secondary forests play an underappreciated role in providing wild foods. Younger recently disturbed forests and those regenerating after disturbance likely support dietary diversity in different ways than older or more intact forests because their function, structure, and composition differ (Brown and Zarin 2013, Tropek et al. 2014, Sutherland et al. 2016, Watson et al. 2018). For example, in Tanzania, wild leafy greens collected from disturbed forests are an important source of nutrition largely unavailable in primary forests (Powell et al. 2013b, Magnago et al. 2015). Many wild foods are found within forest fallows (i.e., young regenerating forests on previously cropped fields; Brookfield and Padoch 1994, Broegaard et al. 2017). Fallows support legacy species from prior cultivation, as well as from previously discarded pits and seeds (Wood et al. 2016), along with intentionally planted species (Sanchez 1999). Fallows and secondary forests are often important sites for hunting (Naughton-Treeves 2002, Smith 2005, Nasi et al. 2011). Initial evidence suggests that landscapes that include fallows and swidden agroforestry are associated with higher consumption of micronutrient-rich food groups than are landscapes with simplified agricultural systems (Ickowitz et al. 2016).

These relationships are complex, however. In the Brazilian Amazon, primary forests could sustainably provide more wild meat (per hectare) than secondary forests (Parry et al. 2009). However in the Bolivian Amazon, the density of useful plant species was lower in mature forests than in secondary forests (Toledo and Salick 2006). In the Peruvian Amazon, young fallows provided fewer useful species than secondary forest, but their total monetary value was greater (Gavin 2004). Finally, tree species composition within planted and regenerating stands likely affects their function, in part, because of the simplified forest structure of some forest plantations and managed secondary forests that have less diversity of tree and understory species (Nájera and Simonetti 2010). As an example, within some Amazonian riparian areas, extensive açai palm forest management has produced monodominant forests (Weinstein and Moegenburg 2004).

### Forest edge habitats as nutritional ecotones

Over 70% of the world's remaining forests are within 1 kilometer of a forest edge (Haddad et al. 2015). Forest edge ecotones—where forests meet other land cover types—consist of altered light, moisture, and nutrient conditions and are characterized by higher species diversity of plants and animals. This fundamental edge effect principle of ecology (Saunders et al. 1991, Haddad et al. 2015) potentially influences the type and amount of forest foods available near forest edges. Species preferring high light environments, such as pioneer or weedy species, thrive at forest edges (Magnago et al. 2015). Guava (Psidium guajava) serves as a prime example; it can be invasive along forest edges but provides an important fruit resource for people and animals (Berens et al. 2008). Forest edges also provide improved access points into forest interiors from which wildlife (for bushmeat) and fuelwood (for cooking) can be extracted.

Forests also affect adjacent agricultural lands (Mitchell et al. 2015) and, in doing so, can indirectly influence dietary diversity by affecting agricultural productivity. Among the most well understood positive influences is the impact of forests on crop pollination (Ricketts et al. 2004, Bailey et al. 2014). Roughly a third of global food comes from pollinated crops, many of which are also nutrient dense (Eilers et al. 2011). Insects at forest edges can enhance pest control (via natural enemies) or result in damaging crop herbivory (Bianchi et al. 2008). Raiding of crops and livestock by forest wildlife can create significant vulnerabilities for food security of rural households, however (Dorresteijn et al. 2014). Other disservices may include negative impacts on agricultural yields through competition for light, water, and nutrients (Akbar et al. 1990, Reynolds et al. 2007, Sida et al. 2018b). On balance, edge effects on agricultural production appear to be positive (González et al. 2016).

Interestingly, the total amount and arrangement of forest edge habitats can alter ecosystem services and disservices provided by forest–agricultural landscape mosaics, and the impacts may be perceived differently among various households (Dorresteijn et al. 2014). Furthermore, the total amount and arrangement of forest edge habitats can function in nonlinear ways to affect ecosystem services in landscapes composed of forest and agriculture (Yang et al. 2020). Because edge influences can permeate forest interiors to a depth of 100 meters or more (Laurance 1997, Chaplin-Kramer et al. 2015), a substantive area of the world's forests is potentially subject to edge influences. Therefore, recognizing trade-offs among ecosystem services and disservices is critical for human well-being (Shackleton et al. 2016, Power 2010), and this challenge may be particularly acute near forest edges.

Access mediates the impact of forests on dietary diversity. Households further from forests and trees may have less diverse diets because they lack routine access to forest foods. As the distance to a forest increases, forest foods are likely to be more costly to obtain (in terms of both time and effort; Baudron et al. 2017). In contrast, close proximity to forests can provide opportunistic access to bushmeat species abundant at the forest edges, whereas fruit-bearing trees planted near villages may attract a variety of animal species (rodents and monkeys; Berens et al. 2008, Sunderland and Rowland 2019). Importantly, people may travel much further or deeper into the forest interior for hunting, fishing, or specialty forest foods (e.g., orchid tubers and mushrooms; Cunningham 2011). Although travel distances and movements can be very site specific and are affected by cultural food preferences and terrain, the distance to forests and trees likely affects consumption of forest foods.

In addition to location, permissions and land rights affect access and therefore mediate the role of forests in influencing dietary diversity. For example, despite the high availability of wild foods in protected forests (Ratsimbazafy et al. 2012), National parks and protected areas with restrictions on access or extraction may result in fewer dietary benefits than accessible communal areas (Sylvester et al. 2016). Similarly, rules governing access and extraction rights around private or community-managed forests can limit the harvest of resources (Robinson and Lokina 2011, Jagger et al. 2014). Importantly in some landscapes, forest resources serve as an economic equalizer, making disproportionate contributions to livelihoods for resource-poor, land-poor, or female-headed households (Kamanga et al. 2009). Therefore, the loss of access may disproportionally affect such households and their diets. Thus, the presence of forest within landscapes may not be a straightforward predictor of improved dietary diversity if the local people do not have access (Naidoo et al. 2019).

Landscape diversity can bolster dietary diversity. When considered collectively, the aforementioned patterns suggest that diverse heterogeneous landscapes may be better equipped to support diverse diets, particularly in rural landscapes in which market access is low. Where local landscapes—landscapes on which people rely—provide reasonable access to the ecosystems and land cover types needed for diverse foods, it is more likely people will have access to diverse diets. For example, in Tanzania, many of the vegetables consumed grow as wild species along forest edges or within fallows (Powell et al. 2013b). Consumption of fruit relies on agroforests, scattered trees, and disturbed or edge forest (Powell et al. 2015). Meanwhile, home gardens often support species not found elsewhere (Powell et al. 2015). In many parts of the world, meat consumption requires access either to large forest tracts with wild game or to areas producing feed or fodder for domesticated animals. Finally, grains, legumes, and some roots or tubers require farmed land.

Simply put, different species and food groups require different ecological niches, but rarely are all these drivers of dietary diversity examined in a unified way. However, such diversity in local landscapes can enhance dietary diversity by providing a variety of nutrient-dense food items in addition to what can be procured in markets. It is precisely in such rural landscapes where forest and biodiversity loss are of concern (Dawson et al. 2019), along with food security and poverty alleviation, further emphasizing the need to clarify the significance of forests in concert with other land cover types.

In addition to landscapes composed of many land cover types, the type and spacing of agricultural fields may also provide an indication of available dietary diversity (Kumar et al. 2015, Herrero et al. 2017,). Larger fields (i.e., clumped, unified parcels) comprising inedible cash crops or monocultures (e.g., palm oil) are typically indicative of specialization. Specialized production systems and monoculture-dominated landscapes are less likely to directly provide diverse dietary resources, especially to local residents. In contrast, landscapes with smaller fields are more likely to include traditional forms of agriculture, involving intercropping, rotations, and mixed crop–livestock production, as well as agroforestry and, therefore, a potentially greater range of agricultural products (Fanzo 2017, Herrero et al. 2017).

The majority of fruits, vegetables, and pulses are produced in more diverse agricultural landscapes (Herrero et al. 2017), and the majority of food in sub-Saharan Africa, Southeast Asia, South Asia, and China is produced in small farms (Herrero et al. 2017). Therefore, there is evidence that small, diverse farms are key for supplying nutrient-rich foods in many regions of the world. Incorporating more complex aspects of landscape diversity, both within and beyond agriculture, takes a step further in generating an understanding of landscape features important in diet diversity. Fully recognizing the role of landscape diversity could help integrate the various aspects of forests, trees, and farms capable of supporting diverse diets.

## A way forward for filling knowledge gaps

Several conceptual and methodological gaps currently stymie our ability to uncover the role of landscape diversity in supporting dietary diversity. We propose several ways forward to help better understand these relationships.

Less compartmentalized approaches are needed to understand the many pathways to dietary diversity. First and foremost, a guiding framework to support transdisciplinary approaches is essential because landscapes can contribute to dietary diversity through many interacting pathways (figure 1), and the understanding of each requires expertise from many disciplines. The direct pathway from forests to dietary diversity (pathway 1 in figure 1) captures the known direct contributions of forests to diets in the form of wild or forest-sourced foods, ranging from fruits and vegetables to fish and bushmeat (Fa et al. 2003, Vinceti et al. 2008, Nasi et al. 2011, Termote et al. 2011, Powell et al. 2013b). In addition, forests also affect dietary diversity via less direct agroecological pathways (pathway 2) through which forests support agriculture. The agroecological pathway includes a wide variety of ecosystem services that support agricultural production (including the maintenance of soil fertility, pollination, and pest control; Ricketts et al. 2004, Bianchi et al. 2006, Reed et al. 2017, Wood et al. 2018). Furthermore, forests serve as a source of feed and fodder for livestock, which then produces animal products for direct consumption (meat, milk, eggs), as well as soil amendments (manure) for row crops and home gardens (Baudron et al. 2017). The fuels pathway (pathway 3) highlights how forests and agroecological pathways can ameliorate energy poverty for households with insufficient energy to cook or for those spending hours on fuelwood collection (Wan et al. 2011, Baudron et al. 2017). Fuelwood from forests, along with dung from livestock, not only facilitates cooking a range of foods for many households but also supports the preparation of nutrient-dense foods with long cooking times, such as legumes (Powell et al. 2015).

Market access can either amplify or counteract the dietary benefits of forests. Finally, the role of income and market access (pathway 4) in supporting higher dietary diversity is complicated in rural forested areas, with important caveats and trade-offs (Pfund et al. 2011, Angelsen et al. 2014). Forest-adjacent communities are often some of the most remote and poorest in a country or region. Despite the many forest products that can be sold to generate income, the importance of income from forest products relative to other sources is mitigated by market access and other factors. The relative importance of income from the sale of forest products may change in times of crises (e.g., drought, illness, or other shocks). Although the sale of forest products can provide rural households with an income safety net (Shackleton and Shackleton 2004, Shackleton et al. 2007, Arnold et al. 2011), other coping strategies may be more common (Wunder et al. 2014). In Honduras, the sale of forest products, although it is not the most common coping mechanism to deal with hurricane related crop loss, was used most commonly by young, poor, and land-poor households (McSweeney 2004).

Whether or not increased market access or income will improve diets depends on aspects of the nearby markets. In rural areas in which local infrastructure (electricity, refrigeration, transportation, etc.) is not well developed, highly perishable foods (such as fresh fruits, vegetables, fish, and meat) do not travel long distances. In such places, markets may only supply locally produced perishable foods, in addition to nonperishable processed foods from regional or global markets (Ickowitz et al. 2019). Because of this, greater market access can be associated with higher access to and consumption of processed foods (Reyes-Garcia et al. 2019), which are micronutrient poor and high in energy, salt, sugar, and fat. Therefore, although markets can enhance dietary diversity by providing access to a wider range of foods, there are complex interactions among forests, market access, and nutrition that have yet to be well understood.

Because the majority of fruits, vegetables, and pulses are produced in diverse agricultural landscapes (Herrero et al. 2017), markets are also key for ensuring that nutrient-rich foods reach consumers outside of such origin or production landscapes. Therefore, markets bring some of the benefits of the nutrient-rich foods from diverse landscapes to other consumers (beyond the landscapes in which the food was produced) in both rural and urban landscapes. To fully appreciate the role of markets, nutrition-sensitive landscapes should be studied as socioecological systems (Kalaba 2014) that involve the choices of local farmers and their relations with other beneficiaries (either directly or indirectly through markets or teleconnections). Such perspectives are critical to understanding the dynamics between rural areas and growing urban centers, inform debates on local versus global food sourcing, and indicate the scale and intensity of land use required to feed the global population with a diversified healthy diet.

## Improvements in future monitoring and research methods

To understand these four pathways and evaluate the strength of evidence supporting or negating each, several monitoring gaps must be filled. Methods for improving the empirical assessment of forest cover and dietary information are explained next.

## Filling gaps in measures of dietary intake

Evaluating the direct contribution of forests to diet (as in pathway 1 in figure 1) requires an understanding of the origin of food products. Unfortunately, several methodological issues surrounding collection of dietary intake data limit our ability to understand the importance of this pathway. First, research on dietary intake does not routinely determine the origin of food, be it nearby forests, a farmer's own fields, or markets. Food from markets may also be of indeterminate origin (fields or forests) or may come from different countries. Because very few studies gather this level of detail, most studies cannot provide direct attribution of forest-sourced foods, much less the type of forest from which a food was collected. Many large, publicly available data sets contain information at the food group level (fruit, meat, dairy, etc.) and therefore lack the species or varietal information needed to trace a food's origin (e.g., https://dhsprogram.com). Such coarse levels of information hinder our understanding of forests’ contribution to nutrition.

Seasonality affects the availability and use of forest foods; therefore, diet diversity can change seasonally (Waswa 2016, Stevens et al. 2017). In Malawi and Zambia, the proportion of women meeting minimum diet diversity requirements fluctuated widely depending on the month of data collection (Ahern and Kennedy 2018). In contrast, dietary diversity did not change across seasons in Tanzania; however, the source of foods did change, whereby wild food consumption was greater during the food-insecure season (Powell et al. 2013b). Although consumption of wild foods is highly seasonal, it is unclear whether this is driven by need or availability (Powell et al. 2015). Seasonal nutritional patterns must be better characterized if we are to understand the contributions of forests to nutrition.

A greater depth and nuance in dietary diversity metrics could also improve our understanding of landscape-diet relationships. Diversity indices are quite well developed and routinely used in ecology to characterize species and land cover diversity, probing well beyond just total species counts. For example, tracking identities (species, cultivars, and varieties) would be useful in determining how local (alpha) diversity is generated. Furthermore, the benefit of using more complex diversity indices would enable analysis of diversity differences among households (such as beta diversity), and their contributions to the total diversity in a landscape (gamma diversity). Lachat and colleagues (2018) used dietary species richness (DSR) to explore diversity beyond food groups, thus capturing the biological diversity of diets. DSR has been validated and shown to be positively correlated with micronutrient intake and diet quality across multiple countries (Lachat et al. 2018). Nutritional functional diversity has also been linked to dietary quality (Lachat et al. 2018) and lower incidence of child malnutrition at the national scale (Remans et al. 2011). Because some such metrics can be challenging to interpret, Wood (2018) developed the potential nutritional adequacy score, a simplified but intuitive indicator capturing multiple dimensions of nutritional diversity. It has been used to assess how different production systems contribute to nutritional needs in Senegal (Wood 2018).

In summary, to better understand the contribution of diverse landscapes to dietary diversity, dietary intake assessments must pay more attention to food origin, seasonal variation, and consider a greater more creative range of diet diversity metrics.

## Improved monitoring and mapping of trees and forests

Advances in remote sensing could help evaluate the attributes of forests, woody vegetation, and scattered trees which are important to diets but are not typically captured in routine forest cover mapping. Several knowledge gaps could be filled by the use of high spatial resolution imagery, a better characterization of seasonal forest phenology, and a clear understanding of historical forest change.

Unfortunately, many definitions of forest used in monitoring not only underestimate tree cover (Chazdon et al. 2016) but potentially underestimate forest types of value to nutrition, as well as to ecosystem services (Gross et al. 2017). As such, the definitions of forest and nonforest used in satellite remote sensing (Chazdon et al. 2016) are important to reconsider in light of forests’ role in food security and dietary diversity. For example, minimum thresholds for tree canopy cover and forest patch size are often used in mapping to delimit an area as forest. Notably, over 40% of agricultural lands worldwide have more than 10% canopy cover (Zomer et al. 2014), coincidentally exceeding the 10% canopy threshold often used to define an area as forest (FAO 2010). Use of 0.5-hectare minimum patch size thresholds (as in http://mapbiomas.org) disregards small forests fragments and remnants, such as sacred forest patches, home gardens, narrow riparian forests, live fences, and scattered fruit trees. Such fine-scale features are generally missed by the spatial resolution of sensors on satellites in routine use historically (such as 30-meter Landsat), making such features difficult to monitor (Gergel 2007). Such forest mapping criteria influence the detection, classification, and characterization of landscape diversity, particularly so in places with sparse tree cover or small forest fragments (Chazdon et al. 2016). Although food resources from small forest patches and scattered trees have largely been overlooked by the development community (Kumar 2006), they are, in addition, simply not well captured in routine forest monitoring.

The use of high spatial resolution satellite imagery (e.g., WorldView-3, Quickbird, SPOT, and RapidEye) can capture individual trees (Li et al. 2017), riparian forests (Johansen et al. 2007), and sparse savanna tree cover (Boggs 2010). Colgan and colleagues (2012) successfully combined high spatial resolution aerial imagery and lidar to map tree species in South African savannas. High resolution imagery has been used to generate settlement maps across several developing countries (e.g., Tatem et al. 2007), which could be used to estimate the occurrence of home gardens. Although the ecological importance of large scattered trees is appreciated (Manning et al. 2006), understanding the role of scattered trees or sparse woody vegetation in nutrition will necessitate the use of higher spatial resolution approaches to capture fine-scale attributes because fruit consumption relies on agroforests, scattered trees, and home gardens (Powell et al. 2015).

Temporal aspects of food–forest dynamics require forest tracking across different timeframes from the short term to the longer term. Throughout much of Africa, vegetation greenness varies dramatically across the year (Zhang et al. 2018) affecting forest resources. For example, in Burkina Faso, edible leaves from trees (such as the baobab tree, Adansonia digitata) provide up to 60% of consumed vegetables. As a deciduous species that loses its leaves in the dry season, its availability to produce food is highly seasonal. As a result, to ensure vegetable consumption in the dry season, people must either dry tree leaves or have access to irrigated gardens (Mertz et al. 2001, Lykke et al. 2002). Such phenological changes present challenges for deriving accurate consistent vegetation information because satellite imagery often provides coverage at either high spatial resolution over infrequent intervals or at frequent intervals but with coarse resolution. Among possible solutions include use of high spatial resolution imagery to train more frequent moderate resolution imagery (e.g., Brandt et al. 2018), as well as the inclusion of mapping targets (such as buildings) that lack phenological variability.

Distinguishing between older forest remnants and newly established forests is not only important for ecological reasons (Chazdon et al. 2016) but, as was previously discussed, is potentially of great importance to nutrition. The structure and composition of new tree cover and younger forests differ from those of older forests (Brown and Zarin 2013, Sutherland et al. 2016). Although many regions of the world now lack large tracts of primary undisturbed forest, many forest assessments do not distinguish between planted and naturally regenerated forests or between stands of different age. Without such distinctions, the expansion of plantations may be portrayed as a gain (or no net loss) of forests (Puyravaud et al. 2010, Chazdon et al. 2016, Petersen et al. 2016). Even Hansen's extremely useful and ambitious map of global deforestation classifies rubber, oil palm plantations, and other monocultures as forest cover (Hansen et al. 2013, Tropek et al. 2014). Gaining a deeper perspective of forest trajectories and how they affect forest foods will necessitate longer-term image time series from sources such as Landsat (Hansen et al. 2013) or historical archival aerial photography (Morgan et al. 2010).

In summary, detailed tracking of forest landscape mosaics over time could be a powerful approach for prioritizing nutrition-based interventions. However, evaluation of nutrition-sensitive landscapes will remain challenging until such mapping is refined.

## Implications for land-use planning, conservation, and forest restoration

Our efforts to theorize and monitor the pathways leading from landscape diversity to dietary diversity can contribute to the goals of several emerging landscape approaches in land-use planning (Sayer et al. 2014, Laestadius et al. 2015) and have implications for conservation, agriculture, and forest restoration, as well as human well-being. Understanding landscape diversity and improving the tools used to measure it will improve our ability to balance the multiple functions and multiple stakeholders that landscapes must support (Sayer et al. 2014).

High spatial resolution mapping and monitoring has the potential to not only aid nutritional planning but also yield benefits to conservation and restoration initiatives often occurring within the same landscapes (Fisher and Christopher 2007), ensuring nutrition and human well-being are not ignored. For example, tracking small linear forests that provide waterway protection or remnant forest patches and scattered trees that support habitat conservation and connectivity are also useful in planning for landscapes that can support diverse diets. High spatial resolution remote sensing needed to inform landscape pattern and connectivity assessments for conservation could simultaneously contribute information about the types of foods potentially available in a landscape.

Over 500 million smallholder farming households rely on their local landscapes for much of their nutrition (Lowder et al. 2016). Furthermore, small farms in diverse landscapes are producing the majority of the world's food, especially in terms of fruits, vegetables, and important micronutrients (Herrero et al. 2017). However, declining farm sizes in many low-income countries (Lowder et al. 2016), along with transitions to large-scale corporate agricultural production, is placing these farming landscapes under increasing pressure. Higher land-use intensity has been associated with lower use of wild foods (Cooper et al. 2018). Therefore, with increasing land-use intensity, the diets of farming households producing the world's food may change irreplaceably (Ickowitz et al. 2019). Finally, in the face of climate variability, landscape diversity may play an increasingly important role in coping with food shortages (Koffi et al. 2016).

Some nutritionally important foods may be more dependent on ecosystem services such as pollination (Gallai et al. 2009, Eilers et al. 2011, Smith et al. 2015), and as a result, land-use change and homogenization could lead to their decline (Reed et al. 2017). Fortuitously, in the case of forest restoration, there is a growing emphasis on landscape approaches. The term forest landscape restoration encompasses a broader view that recognizes diverse options for both forestry and agriculture (Laestadius et al. 2015). The approach looks beyond site-level technical interventions toward balancing multiple benefits and mitigating trade-offs across landscapes (Laestadius et al. 2015). In fact, the IUCN (International Union for Conservation of Nature) has recently recommended that the World Bank consider landscape approaches in reviews of forest policy (Laestadius et al. 2015). Despite the integral importance of forests to diets, forest resources are not well integrated into poverty alleviation or into nutrition strategies (Oksanen and Mersmann 2003, Powell et al. 2015), but the opportunity exists for their improved integration. Therefore, a landscape perspective on nutrition is both timely and commensurate with emerging priorities for forest restoration.

## Conclusions

Understanding the role of landscape diversity in supporting diet diversity, diet quality, and nutrition is a research imperative. Tackling this challenge requires better integration of expertise that spans multiple disciplines and newly available geospatial information to rigorously evaluate landscape–diet relationships. In the present article, we offer a way forward in addressing the complex interactions between landscape diversity and that of human diets in the rural tropics. First, we summarized the state of knowledge regarding diets obtained from forests, trees, and agroforests. We then hypothesized how specific forest types, as well as overall landscape diversity, can function in supporting dietary diversity. In doing so, we built a framework illuminating four pathways (direct, agroecological, energy, and market pathways) connecting forested landscapes to diet diversity. Finally, we offered recommendations to enhance monitoring of human diets and forest cover designed to help illuminate these pathways.

Biodiversity conservation, climate change, land-use change, agriculture, human health, and nutrition are all integrally affected by landscape structure and diversity. The conceptual and technical approaches we have presented can improve the way competing demands for land are contextualized when food security and nutrition are considered along with forest conservation. The nutrition community is increasingly attentive to issues of sustainability in global diets and dietary recommendations (Hirvonen et al. 2019, HLPE 2019, Willett et al. 2019). Commitments such as the United Nations’ decade of action on nutrition note synergies between the goals of global nutrition and conservation. Also highlighted is the need to move away from an overemphasis on increasing production of staple crops and calories without due attention to diet quality, protection of poor farmers, and sustainability. Along with the United Nation's decade of ecosystem restoration for 2021–2030, such growing national and international commitments across research and policy communities demand transdisciplinary and integrated approaches. Therefore, an opportunity exists to use national and global dietary recommendations to improve the sustainability of food production landscapes around the world while also achieving forest conservation solutions. Truly finding balanced solutions for the multiple functions needed from landscapes will require understanding how landscapes shape diverse nutritious diets.

## Author Biographical

Sarah E. Gergel (sarah.gergel@ubc.ca), Jeanine M. Rhemtulla, Laura V. Rasmussen, and Terry C. H. Sunderland are affiliated with the Department of Forest and Conservation Sciences at the University of British Columbia, in Vancouver, Canada. Bronwen Powell and Erica A. H. Smithwick are affiliated with the Department of Geography and BP is also affiliated with the Departments of African Studies and Anthropology at Pennsylvania State University, in University Park, Pennsylvania. Frédéric Baudron is affiliated with the International Maize and Wheat Improvement Center CIMMYT-Southern Africa Regional Office, in Harare, Zimbabwe. Sylvia L. R. Wood is affiliated with the Future Earth Global Hub, in Montreal, Quebec, Canada. Gina Kennedy and Jessica Ranieri are affiliated with Bioversity International, in Rome, Italy. Amy Ickowitz and Terry C. H. Sunderland are affiliated with the Center for International Forestry Research, in Bogor, Indonesia. Matthew E. Fagan is affiliated with the Department of Geography and Environmental Systems at the University of Maryland—Baltimore County, in Baltimore, Maryland. Stephen A. Wood is affiliated with The Nature Conservancy, Arlington, Virginia, and with the School of Forestry and Environmental Studies at Yale University, in New Haven, Connecticut. Jeroen C. J. Groot is affiliated with the Department of Farming Systems Ecology, at Wageningen University and Research, in Wageningen, The Netherlands.
